# Safety of *Streptococcus pyogenes* Vaccines: Anticipating and Overcoming Challenges for Clinical Trials and Post-Marketing Monitoring

**DOI:** 10.1093/cid/ciad311

**Published:** 2023-05-26

**Authors:** Edwin J Asturias, Jean-Louis Excler, James Ackland, Marco Cavaleri, Alma Fulurija, Raj Long, Mignon McCulloch, Shiranee Sriskandan, Wellington Sun, Liesl Zühlke, Jerome H Kim, James B Dale, Andrew C Steer

**Affiliations:** Colorado School of Public Health, University of Colorado, Aurora Colorado, USA; Children’s Hospital, Anschutz Medical Campus, University of Colorado School of Medicine, Aurora, Colorado, USA; Director General’s Office, International Vaccine Institute, Seoul, Republic of Korea; Global BioSolutions, Melbourne, Australia; Anti-Infectives and Vaccines, European Medicines Agency, Amsterdam, The Netherlands; Group A Streptococcal and Rheumatic Heart Disease Team, Telethon Kids Institute, Perth, Australia; Safety and pharmacovigilance, Bill & Melinda Gates Foundation, London, United Kingdom; Department of Paediatrics, Red Cross War Memorial Children's Hospital, University of Cape Town, Cape Town, South Africa; Department of Infectious Diseases, Imperial College, London, United Kingdom; Vaxcellerant LLC, Silver Spring Maryland, USA; South African Medical Research Council, Parowvallei, Cape Town, South Africa; Division of Paediatric Cardiology, Department of Paediatrics, Institute of Child Health, Faculty of Health Sciences, University of Cape Town, Cape Town, South Africa; Director General’s Office, International Vaccine Institute, Seoul, Republic of Korea; College of Natural Sciences, Seoul National University, Seoul, Republic of Korea; College of Medicine, University of Tennessee Health Science Center, Memphis Tennessee, USA; Infection and Immunity Theme, Tropical Diseases Research Group, Murdoch Children's Research Institute, Parkville Victoria, Australia

**Keywords:** safety, vaccine, *Streptococcus pyogenes*, acute rheumatic fever, clinical trial

## Abstract

*Streptococcus pyogenes* (Strep A) infections result in a vastly underestimated burden of acute and chronic disease globally. The Strep A Vaccine Global Consortium’s (SAVAC’s) mission is to accelerate the development of safe, effective, and affordable *S. pyogenes* vaccines. The safety of vaccine recipients is of paramount importance. A single *S. pyogenes* vaccine clinical trial conducted in the 1960s raised important safety concerns. A SAVAC Safety Working Group was established to review the safety assessment methodology and results of more recent early-phase clinical trials and to consider future challenges for vaccine safety assessments across all phases of vaccine development. No clinical or biological safety signals were detected in any of these early-phase trials in the modern era. Improvements in vaccine safety assessments need further consideration, particularly for pediatric clinical trials, large-scale efficacy trials, and preparation for post-marketing pharmacovigilance.

## Teaser

Safety assessment methodology and future challenges for the clinical development of *Streptococcus pyogenes* vaccines are reviewed and discussed. Improvements in vaccine safety assessments, particularly for pediatric clinical trials, large-scale efficacy trials, and preparation for post-marketing pharmacovigilance, need further consideration.

Infections caused by *Streptococcus pyogenes* (Strep A), a human pathogen, afflict more than 800 million people each year and result in an estimated 639 000 deaths, most attributable to rheumatic heart disease (RHD) and invasive infections [1]. Clinical manifestations vary from mucosal diseases (pharyngitis, tonsillitis, superficial skin infections) to locally invasive and systemic diseases (bacteremia, meningitis, puerperal sepsis, necrotizing fasciitis, toxic shock syndrome) and immune-related sequelae including acute rheumatic fever (ARF), acute post-streptococcal glomerulonephritis, and RHD [1, 2]. While most manifestations of *S. pyogenes* infection can be treated with penicillin, the lack of prevention strategies, particularly in low- and middle-income countries (LMICs), leads infection to progress to severe disease and death. Hence, the 71st World Health Assembly asked the World Health Organization (WHO) in May 2018 to prioritize the development of a safe and effective *S. pyogenes* vaccine.

## PURPOSE OF THE SAVAC VACCINE SAFETY WORKING GROUP

The path to *S. pyogenes* vaccines has been delineated by the WHO Research and Development Technology Roadmap and Preferred Product Characteristics (PPC), with emphasis on vaccine safety considerations and the need to build consensus [3]. The Strep A Vaccine Global Consortium (SAVAC) was established in 2019 and convened experts from the health and research sectors to facilitate the development of *S. pyogenes* vaccines [4]. The overall objective of the SAVAC Safety Working Group was to provide, in a nonprescriptive manner, key vaccine safety considerations for vaccine developers, clinicians, and regulatory authorities. Four subworking groups were constituted to address *S. pyogenes* infectious and post-infectious immune pathogenesis and research into immune markers; the current state of knowledge about *S. pyogenes* vaccine safety; regulatory perspectives; and safety monitoring in phase 1/2 and phase 3 clinical trials and consideration for post-marketing pharmacovigilance.

## THEORETICAL CONCERNS ABOUT VACCINES RELATED TO RHEUMATIC FEVER PATHOGENESIS

The pathogenesis of *S. pyogenes* infection is highly complex, and the mechanisms that lead to autoimmune diseases such as ARF and RHD remain elusive [5]. It has been proposed that certain *S. pyogenes* antigens are involved in cross-reactivity and pathogenesis of ARF, but the evidence to implicate individual antigens is limited and inconclusive. The primary antigen proposed to generate cross-reactive antibody is the surface M protein, encoded by the *emm* gene, a highly variable protein with more than 200 sequence variants (*emm* types) [6]. Some of the monoclonal antibodies generated against a single M-protein variant have been shown to bind human cardiac myosin in heart tissue [7, 8]. Experimental proof that M protein is the ARF antigen is lacking. The absence of adequate models for ARF/RHD represents a major limitation to research in this regard. In addition, there are indications that antibodies to the *S. pyogenes* group A carbohydrate can bind to human heart tissue and may be involved in ARF pathogenesis [8, 9]. Non-M proteins such as hyaluronic capsule and 2 proteins present in the *S. pyogenes* cell membrane were also identified as cross-reactive antigens [10, 11]. These theoretical concerns have been raised during the development of *S. pyogenes* vaccines based on M protein or group A carbohydrate.

## STATE OF KNOWLEDGE ABOUT *S. PYOGENES* VACCINE SAFETY

There is a long history of vaccine trials for *S. pyogenes* in humans, dating back more than 100 years and involving more than 200 000 participants. One trial, conducted by Massell and colleagues in the United States in the 1960s, raised safety concerns, including with US regulators [12, 13]. Over the past 2 decades, there have been 5 *S. pyogenes* vaccine clinical trials conducted. Here, we review the Massell trial and the approach and results of safety assessments for the 5 trials in the modern era.

### The Massell Trial

The Massell study was conducted between 1965 and 1967 in Boston [12, 13]. The vaccine was a hot-acid extracted M protein of a type 3 *S. pyogenes*, partially purified using ribonuclease and dissolved in thiomersal. Study participants were 21 healthy siblings of patients with a history of ARF, randomly selected from a cohort of 106 healthy siblings. There was no control group. The 21 children were given weekly subcutaneous injections of gradually increasing doses of the vaccine, necessary because of reactogenicity, for 18 to 33 weeks.

**Figure 1.**
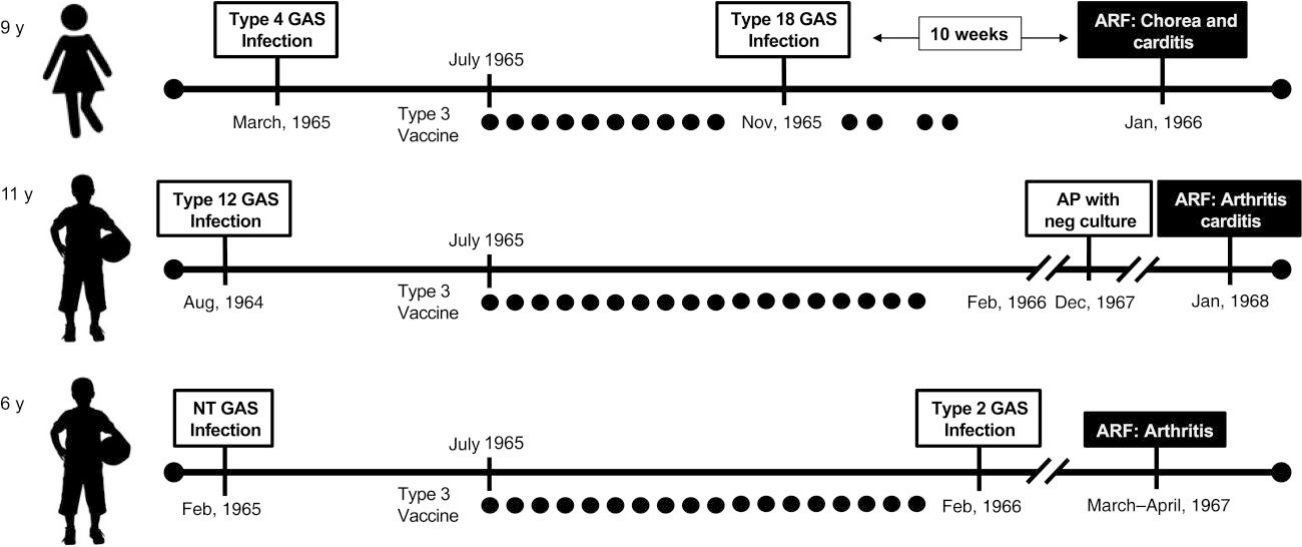
Pictorial representation of 2 cases of definite rheumatic fever and 1 case of probable rheumatic fever after vaccination with a hot-acid extracted M protein of a type 3 *Streptococcus pyogenes* partially purified using ribonuclease and dissolved in thiomersal. Dots represent vaccine dose). Abbreviations: ARF, acute rheumatic fever; GAS, Group A Streptococcus ; NT, not typeable).

The vaccination program began in July 1965 with a 30-month observation period. There were 18 episodes of *S. pyogenes* pharyngitis (none were type 3; Figure 1). One child (female aged 9 years) developed chorea and a grade 3 pansystolic murmur. This child had a documented infection with emm18 *S. pyogenes* prior to her ARF illness, a strain type epidemiologically linked to ARF [14]. Another child (male aged 11 years) developed fever, right shoulder pain, right knee arthritis, and carditis (pansystolic murmur, diastolic murmur, and heart block). A third child (male aged 6 years) developed fever and arthritis in both knees. The authors diagnosed definite ARF in the first 2 children and probable ARF in the third child. No data were provided on episodes of ARF in the 85 other healthy siblings. The authors compared the number of ARF episodes as a proportion of cases of sore throat (3 of 18, 17%) with a historical cohort of nonvaccinated children (all siblings of ARF patients) observed over a 15-year period. In this historical cohort of an unspecified number of children, there were 447 episodes of *S. pyogenes* pharyngitis and 5 cases of ARF (1%). The statistical comparison of these 2 groups had a *P* value of <.001. The authors concluded with “the need for extreme caution in conducting studies with streptococcal vaccine in human participants” [13].

Despite the flaws of the study design, this potential safety concern contributed to the US Food and Drug Administration (FDA) regulation enacted in January 1979. As part of an efficacy review of all biologicals approved prior to 1972, the FDA convened a Panel on Review of Bacterial Vaccines and Bacterial Antigens (with no U.S. Standard of Potency) to review “mixed bacterial vaccines.” Reviewing the Massell study and noting ARF cases following vaccination, the panel concluded that uncontrolled use of *S. pyogenes* antigens in bacterial vaccines with “no U.S. standard of potency” represented unacceptable risks, and the FDA Commissioner codified this conclusion in 21 CFR 610.19, Status of specific products: Group A *Streptococcus* [15]. The regulation was revoked from the US *Federal Register* in December 2005 (Box 1) as the FDA believed that its requirements for *S. pyogenes* organisms and derivatives were “both obsolete and a perceived impediment to the development of Group A streptococcus vaccines” [16].

Box 1. **Revocation of the 21 CFR 610.19, Status of specific products: Group A *Streptococcus*, 2 December 2005 [16].**

“We are removing § 610.19 because the existing requirement is obsolete and perceived to be impeding the development of Group A streptococcal vaccines using purified or characterized streptococcal antigens. The regulation is obsolete because it was written to apply to a group of products that are no longer on the market. Therefore, a vaccine to prevent diseases caused by this organism would have a public health benefit. We are taking this action as part of our continuing effort to reduce the burden of unnecessary regulations on industry and to revise outdated regulations without diminishing public health protection.”

### Safety Assessment in Vaccine Clinical Trials in the Modern Era

Since the lift of the FDA ban, there have been 5 *S. pyogenes* vaccine clinical trials conducted (Table 1) using antigens from M protein [17]. To address the historical and hypothetical concern of autoimmune responses elicited by antigens derived from *S. pyogenes*, several specific safety assessments were introduced into clinical protocols (Table 2). Standard clinical echocardiographic Doppler-flow examinations were performed in all clinical studies to monitor for rheumatic cardiac abnormalities prior to entry and following final vaccination. The echocardiograms were evaluated independently by 2 cardiologists; when disagreement occurred, a third independent assessment was obtained. Criteria were used to evaluate pre- and post-vaccination echocardiograms using predetermined structural and functional changes.

**Table 1.** Summary of the Vaccine Product Characteristics, Dosing Regimens, Population, and Designs Used in Recent *Streptococcus pyogenes* Vaccine Clinical Trials, 1990–2020

Trial	Product	Dose Regimen	Control	Population	N	Design	Regulatory Agency
Adult phase 1 [18]	Hexavalent prototype; N-terminal peptides from M1, 3, 5, 6, 19, and 24	Successive cohorts received: 50 µg IM on d 0, 28, and 56 (N = 8) 100 µg IM on d 0, 28, and 112 (N = 10) 200 µg IM on d 0, 28, and 112 (N = 10)	None	Healthy adults aged 18–50 y	29	Open-label, dose-escalation	US Food and Drug Administration
Adult phase 1 [19]	StreptAvax 26-valent, N-terminal M peptides	400 µg IM on d 0, 28, and 120	None	Healthy adults aged 18–50	30	Open-label	Health Canada
Adult phase 2 [20]	StreptAvax 26-valent	400 µg IM on d 0, 28, and 180	Hepatitis A vaccine	Healthy adults aged 18–50	90	Randomized, double-blind, comparator-controlled (70 StreptAvax, 20 comparator)	Health Canada
Adult phase 1 [21, 22]	StreptAnova 30-valent, N-terminal M peptides	600 µg IM on d 0, 28, and 180	Selected licensed vaccines	Healthy adults aged 18–50	36	Randomized, double-blind, comparator-controlled (23 StreptAnova, 13 comparator)	Health Canada
Adult phase 1 [23]	MJ8VAX (J8-DT) C-terminal 29 aa M peptide	50 µg IM on d 0	Saline	Healthy adults aged 20–44	10	Randomized, double-blind, placebo-controlled (8 MJ8VAX, 2 placebo)	Queensland Institute of Medical Research Human Research Ethics Committee

Abbreviation: IM, intramuscularly.

**Table 2.** Safety Assessments Performed in Recent *Streptococcus pyogenes* Vaccine Trials

Hexavalent Prototype Multivalent M [18]	26-Valent (Phase 1) Multivalent M [19]	26-Valent (Phase 2) Multivalent M [20]	30-Valent (Phase 1) Multivalent M [21, 22]	J8-DT Conserved C-Terminal M Peptide Conjugate [23]
7-d reactogenicity diary Cardiac exam 14 d after each dose, and 6 and 12 mo Echocardiogram and ECG 14 d after each dose, and 6 and 12 mo Routine clinical labs + troponin, C3, CRP Human tissue cross-reactive antibodies by IFA 14 d after each dose, and 5 and 12 mo AE follow-up × 12 mo	14-d reactogenicity diary Cardiac and neurologic exams 7 and 14 d after each dose Echocardiogram and ECG screening and within 1 month after third dose Routine clinical labs plus troponin-I, C3, CRP Human tissue cross-reactive antibodies by IFA within 1 month after second and third doses AE follow-up × 12 mo	14-d reactogenicity diary Cardiac and neurologic exams 7 and 14 d after each dose Echocardiogram and ECG screening and within 1 month after third dose Routine clinical labs plus Troponin-I, C3, CRP baseline and if indicated to evaluate clinical findings Human tissue cross-reactive antibodies by IFA within 1 month after second and third doses AE follow-up × 12 mo	14-d reactogenicity diary Cardiac, neurologic, and joint exams 7 d after dose 1 and 14 d after doses 2 and 3 Echocardiogram and ECG screening and 30 d after third dose Routine clinical labs plus C3, CRP baseline and if indicated to evaluate clinical findings Human tissue cross-reactive antibodies by IFA screening and 14 d after each dose AE follow-up × 12 mo	7-d reactogenicity diary Cardiac exam d 28, 180, 266, and 350 Echocardiogram and ECG screening and d 28, 84, and 350 Routine clinical labs screening and d 28, 84, 180, 266, and 350 Serum stored pretreatment and d 350 for future tissue cross-reactive antibody assays AE follow-up × 12 mo

Abbreviations: AE, adverse event; CRP, C-reactive protein; ECG, electrocardiogram; IFA, immunoflorescence assay.

There have been no serious safety signals detected among these 5 trials (Supplementary Table 1). No participant developed clinical, echocardiographic, or laboratory evidence of rheumatogenicity or nephritogenicity. No induction of human tissue–reactive antibodies was demonstrated in the first 4 studies; they were not measured in the fifth.

## REGULATORY PERSPECTIVES TO INFORM SAFETY STRATEGIES

Further development of *S. pyogenes* vaccines requires safety as a primary outcome to be agreed on by developers and relevant national regulatory authorities (NRAs). The details of specific safety requirements for licensure may vary due to applicable national laws and product characteristics of the particular investigational vaccine. Currently, no specific requirements for *S. pyogenes* vaccine development are in place by the FDA or other regulatory agencies. General requirements for vaccines for other infectious diseases should be used as a starting point for consideration. However, because of the specific considerations that would be required for *S. pyogenes* vaccines, early, close, and periodic consultation with regulatory agencies throughout the life cycle of development for *S. pyogenes* vaccines will be necessary (Box 2).

Box 2. **Reasons for close and periodic consultation with regulatory agencies throughout the life cycle of vaccine development for *S. pyogenes* vaccines.**

1. Unique considerations for *S. pyogenes* vaccines, including the potential for vaccine-induced immune-mediated sequelae
2. Rigorous evaluation of new nonclinical and clinical data for novel vaccines with similarities to *S. pyogenes*
3. Evolution of new technologies used in product development
4. Available and expected testing capacity, including assay development and evaluation of immune and safety responses
5. Evolution of risk–benefit assessment as part of product evaluation and risk management plans
6. Specific pharmacovigilance commitments and phase 4 studies
7. Assessment of potential public health impact, particularly for a vaccine for which efficacy may be variable according to clinical outcomes (eg, partial protection against pharyngitis but higher efficacy against immune-mediated sequelae).

### Preclinical Safety

There is currently no specific regulatory consensus on an adequate preclinical assessment of potential vaccine-induced autoimmunity before the first-in-human study. Harmonization of a core set of preclinical safety data among several NRAs could provide a standardized process of characterization of vaccine-induced immune responses and safety evaluation to accelerate development. Specific studies that use modern technologies should be informed by the current understanding of the pathogenesis of *S. pyogenes* immune-mediated disorders and the validity of animal models as applied to humans, being careful to acknowledge any uncertainty and knowledge gaps. In vitro tissue cross-reactivity studies have been used routinely in preclinical safety evaluation of other biologics such as monoclonal antibodies to study on-target and off-target tissue binding [24]. Their role and relevance in *S. pyogenes* vaccine development are uncertain; any tissue cross-reactivity assays to detect cardiac signals would require proof of biological plausibility, validation, and standardization. The choice of the most relevant in vivo animal model for preclinical safety evaluation should be discussed with the regulator at the earliest opportunity.

### Prelicensure Clinical Safety

Clinical trials should be conducted first in healthy adults to determine the baseline safety and immunogenicity profile. As children and adolescents bear the major burden of ARF, once safety and immunogenicity have been demonstrated in adults, sequential phase 1/2 trials with proportionate deescalation in age to the target vaccine population of younger children should be conducted before the full phase 3 demonstration of safety and efficacy in that population. The dose and regimen selected for the pivotal phase 3 placebo-controlled efficacy trial will be based on the safety and immunogenicity of phase 2 dose-finding.

While ARF/RHD may occur after *S. pyogenes* infection, they may potentially occur in a vaccine trial as vaccine-induced autoimmune phenomena, with or without *S. pyogenes* infection. In highly endemic areas, background ARF incidence ranges from 8 to 50 cases per 100 000 population and rarely up to 250 [25]. Thus, even if prevention of ARF/RHD is not a primary end point in the trial, they must be captured as adverse events following immunization (AEFI). While the use of the Jones criteria [26] is sufficient for clinical diagnosis of ARF/RHD, case definitions and ascertainment should be discussed with the regulatory authority prior to the initiation of clinical trials. A data monitoring committee could monitor in real-time any statistically and clinically significant imbalance of ARF/RHD as a safety signal and apply pause rules where appropriate.

The optimal duration of clinical trials, driven by the need to demonstrate both safety and efficacy for a particular vaccine, is important for discussion with regulatory authorities. Importantly, ARF occurs within 2–4 weeks of *S. pyogenes* pharyngitis. However, clinically detectable RHD can have a much longer time to onset, highlighting considerations around duration of safety follow-up and around the role of echocardiography for detecting clinically silent RHD. Although efficacy trials will use symptomatic disease end points, careful consideration should be given to evaluation of the vaccine’s effect on asymptomatic infection during the trial and the effect of asymptomatic infection on the development of ARF/RHD. The trial design should include sound methods to assess the role of *S. pyogenes* carriage (prospectively or retrospectively) in vaccine efficacy, given the variable immune responses observed with pharyngeal carriage among children [27, 28].

### Post-Marketing Safety

As has been evident from coronavirus disease 2019 (COVID-19) vaccine implementation, systematic monitoring of vaccine safety is a continuing process that should be jointly performed with regulatory and public health immunization authorities with a prospectively agreed safety plan. It will be important to develop observational study designs and tools for the evaluation of *S. pyogenes* vaccine safety signals in a larger and more heterogenous population than investigated in preapproval clinical trials.

A risk management plan needs to be developed [29] when the vaccine is submitted for licensure. Increasingly, vaccine manufacturers incorporate a benefit–risk analysis to support policy decision-makers [30, 31]. *Streptococcus pyogenes* vaccines are likely to be licensed and developed in high-income countries and deployed in LMICs. While LMICs are most likely to benefit from a vaccine when considering invasive disease and RHD, preventing harm in vulnerable populations emphasizes the prominent role of ethics committees in this process.

Strong pharmacovigilance systems in LMICs to detect, analyze, and act on adverse events are essential to the safe scaling of new interventions. A demonstrated favorable track record of safety during larger deployment is essential to maintain trust. Early and proactive engagement with organizations such as the WHO, regional public health authorities such as the Africa Centres for Disease Control and Prevention, regional technical advisory groups on immunization, in-country immunization programs, and regulators in LMICs would foster accelerated access to *S. pyogenes* vaccines.

## SAFETY MONITORING ASSESSMENT CONSIDERATIONS FOR FUTURE CLINICAL TRIALS

The 4 phase 1 trials and 1 phase 2a trial of *S. pyogenes* vaccines conducted to date provide a basis for discussion of safety assessments and monitoring in early-phase studies. There have been no phase 2b or phase 3 trials of *S. pyogenes* vaccines in the modern era.

While generic core safety assessments would not fundamentally differ from any other new vaccine for AEFI using the WHO Causality Assessment Algorithm [32], the evaluation of adverse events of special interest (AESIs) specific to *S. pyogenes* may pose more difficulties, in particular, for efficacy studies in children and phase 3 and post-marketing pharmacovigilance studies, underscoring the current gaps in safety assessment methodology. In particular, *S. pyogenes* safety concerns are more “syndromic” in nature and may not be captured and reported into passive reporting systems with discrete diagnoses but rather a cluster of signs and symptoms. Current approaches to safety surveillance rely on analyses of individual MedDRA (Medical Dictionary for Regulatory Activities) adverse event codes. Furthermore, observational studies are performed using predefined discrete case definitions and are most useful for events with clear onset and duration. Finally, safety surveillance infrastructure in LMIC settings that largely use WHO–AEFI causality algorithms may not be optimal for the timely detection of rare safety signals.

### Cross-Reactive Antibody Assays

In the vaccine phase 1 trials outlined above, testing for cross-reactive tissue antibodies by immunofluorescence against human cadaver heart, kidney, cartilage, basal ganglia, and cerebral cortex was performed preenrollment with a negative test required for participant inclusion. Repeat cross-reactive tissue antibody determinations were performed 2 weeks after the second and third vaccinations, respectively. The methods are laborious and not standardized. Consideration should be given to drawing together an expert group to develop antigen-antibody binding assays using a mixture of suspected human cross-reactive proteins with predefined normal ranges.

### Clinical Assessment

The assessment of ARF events in recent trials of *S. pyogenes* vaccine candidates emphasizes the importance of detailed history and periodic physical exams. Clinical assessment should be general, with a focus on cardiac, neurologic, renal, and rheumatologic systems. Because of the nonspecific nature of laboratory testing for ARF, it is critical that laboratory results be interpreted in the context of clinical findings.

### Safety Biomarkers

There are several limitations to the use of biomarkers for safety of *S. pyogenes* vaccines, including no well-defined immune markers that could act as a risk surrogate of ARF development; gaps in knowledge of mechanistic correlates of ARF/RHD development to aid biomarker identification; a lack of clear understanding of the biologic time windows for sequelae of *S. pyogenes* infection to inform safety assessment protocols; and the Jones criteria that are imperfect as a gold standard.

### Echocardiography

The sensitivity of echocardiography to identify RHD during community surveillance is 3 times greater than that achieved by careful clinical examination alone [33]. However, issues remain with the absence of gold-standard echocardiography criteria for subclinical RHD and for an optimum management strategy for patients with clinically silent, mild valvular abnormalities [34]. A large-scale study conducted in Uganda on secondary antibiotic prophylaxis to prevent progression of latent RHD among children highlighted the feasibility and limitations of screening echocardiograms [35]. These difficulties are particularly relevant for surveillance and detection of safety signals. Anticipation of RHD background rates in different regions could provide vaccine developers, regulators, and public health decision-makers with a better interpretation of any safety signal in late-phase studies and an assessment of the potential benefits of a protective vaccine. Examples of these anticipatory safety outcome background rates have been carried out for infant vaccines [36, 37], human papillomavirus vaccines in adolescents [38], influenza H1N1 [39], and COVID-19 vaccines [40]. Therefore, population-based estimates of age-related risk of potential AESIs will be essential before phase 3/4 studies are conducted [41, 42].

Consensus is needed to identify and define the major *S. pyogenes* efficacy end points that will drive future evaluation and use of *S. pyogenes* vaccines. SAVAC has been developing case definitions of *S. pyogenes* disease end points and produced a suite of standardized “best practice” surveillance protocols [43]. In addition, for safety end point evaluations, working with available Brighton collaboration definitions would provide standardized tools for clinical trials and post-marketing surveillance of *S. pyogenes* vaccines.

## CONCLUSIONS

The ultimate decision on the use of a vaccine for individual and public health impact rests on the morbidity and mortality that can be prevented or modified against the safety profile of the vaccine. Whereas the immediate local and systemic reactogenicity of *S. pyogenes* vaccines could be tolerable and transient, there remains concern over the hypothetical induction of adverse post-vaccination immunological responses.

The details of specific safety requirements for licensure may vary with applicable national laws and PPC of a particular investigational vaccine and target population. The field acknowledges that there are challenges to safety surveillance and monitoring, as well as interpretation of potential safety signals across the full clinical vaccine development pathway. There is a clear role for the international expert community to contribute to filling these gaps.

## RECOMMENDATIONS

The standardization of safety outcome measures for *S. pyogenes* vaccines will be a critical next step for the field. Working groups are needed for the following areas to inform safety assessments: clinical trial design, safety and efficacy end point definitions, screening assays for cross-reactivity, role of echocardiography, and biomarker evaluation. Constituting these groups under the umbrella of SAVAC alongside relevant stakeholder engagement will accelerate progress toward vaccine access to curb the impact of this globally significant pathogen.

## Supplementary Data

Supplementary materials are available at *Clinical Infectious Diseases* online. Consisting of data provided by the authors to benefit the reader, the posted materials are not copyedited and are the sole responsibility of the authors, so questions or comments should be addressed to the corresponding author.

## Supplementary Material

ciad311_Supplementary_Data

## Notes

***Author contributions.*** E. J. A. and J. L. E. contributed equally to the design and writing of the manuscript; J. A., A. F., and S. S. contributed to the immunological considerations; M. C. and W. S. contributed to the regulatory considerations; M. M., L. Z., J. D., and A. C. S. contributed to the clinical and echocardiography considerations; and J. H. K. and A. C. S. and all other authors reviewed and approved the manuscript.

**Acknowledgments.** The authors thank the members of the Strep A Vaccine Global Consortium Executive Committee (SAVAC; https://savac.ivi.int/about) for their constant support and input.

***Financial support.*** This work was supported by a grant from the Wellcome Trust awarded to the International Vaccine Institute (IVI) (215490/Z/19/Z). A. C. S. as co-chair of SAVAC did not receive any funding from SAVAC.
