# Supplementary material for: Safety of Streptococcus pyogenes Vaccines: Anticipating and Overcoming Challenges for Clinical Trials and Post-Marketing Monitoring
Source: Clin Infect Dis. 2023 May 26;77(6):917–24. doi: 10.1093/cid/ciad311 (PMC10506775; doi:10.1093/cid/ciad311)
Supplement: ciad311_Supplementary_Data [file ciad311_supplementary_data.docx]

**Supplementary Table1.** Summary of the findings of safety assessments performed in recent S. pyogenes vaccine clinical trials

| **Trial** | **Product** | **N** | **Adverse events (AEs) in vaccinees** | **Echocardiogram** | **Laboratory** | **Cross-reactive antibodies** |
| --- | --- | --- | --- | --- | --- | --- |
| Hexavalent Phase I [38] | Hexavalent Prototype; N-terminal peptides from M1,3,5,6,19 & 24 | 29 | Mild local reactions in 6/29 subjects (29%) within 7 days post 1^st^ injection and 12/28 (43%) within 28 days post second and third injections. One subject with moderate reaction post 1^st^ dose at 50µg. No increase with higher dose. One with low-grade fever post 2^nd^ dose at 200µg associated with rhinorrhea and myalgia. No other symptoms. | Not performed | One African American participant with absolute neutrophil count decreased to 900 cells/μL, 28 days after receiving a single 100-μg dose of vaccine that he tolerated well. He had borderline abnormal serum C3 at this time (92 mg/dL; normal range, 90-180 mg/dL), and intermittent neutropenia (range, 1000-2500 cells/μL) and mildly depressed to low normal serum C3 levels that predated his first injection and continued throughout his 12-month observation period. His clinical course was benign except for intermittent fatigue and arthralgia attributed to a night job. The final diagnosis was benign ethnic neutropenia, not vaccine-related. | None detected |
| Adult  Phase I [39] | StreptAvax  26-valent, N-terminal M peptides | 30 | Mild and self-limited local injection-site reactions reported in the 14-day period after immunization: tenderness and pain on movement of the arm, each reported by up to 70% of subjects and tending to become more frequent after later doses.  Headache (40%–53% of subjects) and tiredness (17%–23%). Up to 27% of subjects reported either nausea or vomiting following the receipt of 1 or more vaccine doses. Fever was uncommon. Sore joints and muscle aches were mild and uncommon, occurring in only 3%–7% and 13%–17% of subjects, respectively. Not associated with objective physical findings, and all were self-limited; none was suggestive of acute rheumatic fever.  No vaccine-related serious adverse events or other AEs suggestive of rheumatogenicity or nephritogenicity occurred. | No changes in electrocardiogram or echocardiogram findings that were suggestive of acute rheumatic fever. | No significant biochemical or hematological abnormalities observed. No subject developed clinically significant proteinuria, hematuria, or red blood cell casts. | None detected |
| Adult  Phase II [40] | StreptAvax  26-valent | 90 | No vaccine-associated serious adverse events. Most AEs were at the injection site and were mild and self-limited. Systemic AEs were uncommon and did not differ between Strep A vaccine group vs and Havrix™ control group. | No changes in ECG or echocardiogram findings that were suggestive of acute rheumatic fever. | No subject developed clinical or laboratory evidence of rheumatogenicity or nephritogenicity. | None detected |
| Adult  Phase I [42] | StreptAnova  30-valent,  N-terminal M peptides | 36 | No adverse events were reported in the 60-minute period after immunization.  No serious adverse events were reported throughout the study.  No evidence of an increase in the severity of solicited reactions with increasing dose number in either treatment group. A difference  observed between the solicited generalized muscle ache reactions  after dose 2 of the Strep A vaccine recipients and comparator recipients  was found to be statistically significant (44.0% vs. 0.0%). All local reactions were graded by participants as mild  (30 mm or easily tolerated), with one exception. One recipient presented a severe injection redness (>100 mm) lasting for more than 48 h after the third dose beginning Day 3 which  resolved by Day 5.  The most frequent systemic complaints  were drowsiness (38.5% of all participants after the 1^st^ dose), nausea (23.1% of all participants after the 1st dose), and generalized muscle aches (20.5%); most complaints resolved after two days post-vaccination. Fever was reported in two participants  (Grade 1 in Strep A vaccine post dose 2 and Grade 3 in comparator group after dose 3). No participant experienced unexplained or persistent fever, arthritis, carditis, subcutaneous nodules, erythema marginatum, or glomerulonephritis.  Unsolicited AEs was similar in both groups after each dose and decreased.  slightly with increasing dose number. 19/26 (73%) recipients of the Strep A vaccine and 7/13 (54%) of the  comparator group reported unsolicited AEs within 28 days after the 1st dose, while 12/25 (48%) Strep A vaccine recipients and 4/13 (31%) participants that received a comparator vaccine reported unsolicited AEs after the 2^nd^ dose, and 8/23 (35%) that received Strep A vaccine and 5/13 (39%) that received a comparator vaccine (HepA, HepB, HPV, all with alum) after the 3^rd^ dose. | No clinically significant changes  in echocardiograms or ECG 30 days post-dose 3 compared to entry exams. | Seven participants had abnormalities  deemed clinically significant between doses 1 and 2: six from the Strep A vaccine group, including one with increased AST, two with decreased neutrophils/white cell count, one with pyuria and two with proteinuria. One control participant had proteinuria. Three participants had lab abnormalities deemed clinically significant after dose 3: one with proteinuria, one with elevated C-reactive protein (both Strep A vaccine recipients) and one comparator vaccine recipient had intermittent hematuria at several visits including baseline, with fewer than 10 red blood cells per high power field but ranging on dipstick from trace to large blood. After review with a nephrologist, the hematuria in this case was deemed not to be clinically significant and did not require further investigation.  Three participants had episodes of proteinuria throughout the study period, one in the comparator group and two in the Strep A vaccine group. Because proteinuria was present prior to Dose 1 in both Step A vaccine recipients, it was felt not likely to be due to vaccine by the investigators. | None detected |
| Adult  Phase I [43] | MJ8VAX  (J8-DT)  C-terminal 29 aa M peptide | 10 | 13 AEs: 2 associated to vaccine: one with headache and one with abdominal pain | No changes in echo-cardiography examinations or ECG findings. | No clinically significant changes in laboratory values were observed.  No changes in anti-streptococcal antibodies | Not done |
